# Beyond the Spike Glycoprotein: Mutational Signatures in SARS-CoV-2 Structural Proteins

**DOI:** 10.3390/idr17060150

**Published:** 2025-12-18

**Authors:** Emil Tonon, Riccardo Cecchetto, Virginia Lotti, Anna Lagni, Erica Diani, Asia Palmisano, Marco Mantoan, Livio Montesarchio, Francesca Palladini, Giona Turri, Davide Gibellini

**Affiliations:** 1 Department of Diagnostic and Public Health, Division of Microbiology, University of Verona, 37134 Verona, Italy; emiltonon@gmail.com (E.T.); riccardo.cecchetto@univr.it (R.C.); virginia.lotti@univr.it (V.L.); anna.lagni@univr.it (A.L.); asia.palmisano@univr.it (A.P.); davide.gibellini@univr.it (D.G.); 2 UOC Microbiology, AOUI Verona, 37134 Verona, Italy; giona.turri@aovr.veneto.it; 3 Department of Diagnostic and Public Health, Division of Hygiene and Preventive, Environmental and Occupational Medicine, University of Verona, 37134 Verona, Italy; marco.mantoan@studenti.univr.it; 4 Hospital Management, University Hospital of Verona, 37126 Verona, Italy; livio.montesarchio@aovr.veneto.it (L.M.); francesca.palladini@aovr.veneto.it (F.P.)

**Keywords:** SARS-CoV-2, whole genome sequencing, structural protein, mutational analysis, genomic surveillance

## Abstract

**Background:** The continuous emergence of SARS-CoV-2 variants represents a major public health concern. Next-generation sequencing (NGS) enables genomic surveillance, facilitating the detection and monitoring of mutations that impact viral evolution. **Methods:** In this study, full-length SARS-CoV-2 genomes were analyzed between February 2022 and March 2024 as part of routine genomic surveillance conducted in Verona, Italy. Mutations in the envelope (E), membrane (M), and nucleocapsid (N) structural proteins were investigated. Only substitutions with a total prevalence of greater than 1% in the study dataset were considered. **Results:** A total of 178 mutations were identified across the three proteins (E: 16; M: 33; N: 129), of which 18 met the inclusion threshold (E: 3; M: 5; N: 10). Mutations were classified according to temporal dynamics as fixed, emerging, or transient. Throughout the study period, fixed mutations were consistently prevalent, emerging mutations appeared later but persisted with an ascending trend, while transient mutations displayed a single frequency peak before disappearing. Several mutations were reported with potential structural or functional relevance based on the existing literature, while others remain of unknown significance. **Conclusions:** The mutational patterns detected in this study broadly reflect global evolutionary trends of SARS-CoV-2. These findings emphasize the importance of continued genomic surveillance and underline the need for integrated experimental approaches to clarify the biological and epidemiological impact of poorly characterized mutations.

## 1. Introduction

As of June 2025, the Severe Acute Respiratory Syndrome Coronavirus 2 (SARS-CoV-2) has infected more than 778 million people and caused nearly 7 million deaths according to the World Health Organization (WHO, https://www.who.int/ (accessed on 25 September 2025).

SARS-CoV-2 belongs to the *Betacoronavirus* genus of the *Coronaviridae* family, with a single-stranded positive-sense RNA genome of approximately 30,000 nucleotides. The SARS-CoV-2 genome encodes four structural proteins: spike (S), envelope (E), membrane (M) and nucleocapsid (N), 16 non-structural proteins (NSPs), and seven accessory proteins: ORF3a, ORF3c, ORF6, ORF7a, ORF7b, ORF8 and ORF9b [1,2].

The S protein is considered the most relevant determinant of viral entry and the principal epitope for immune system recognition [3]. Variants and subvariants lineage classification is mainly based on S mutations and modifications [4]. These variations have the potential to enhance or sometimes decrease viral fitness and may lead to immune evasion of SARS-CoV-2 from monoclonal antibodies, reducing vaccine effectiveness [5,6]. In spite of the importance of the S viral protein, the genome and protein mutations detected in the other structural proteins may also be involved in the viral evolution and have a clinical impact.

The E protein, comprising 75 amino acids, plays a pivotal role in virus assembly. It is a viroporin and forms pentameric pores by self-assembly, which are involved in ion transport. However, the E protein can also be found in a monomeric form [7,8], which is involved in virion packaging and, together with M, slows host secretory pathways, retaining glycoproteins, including the protein S [2].

Similarly to SARS-CoV, the SARS-CoV-2 Wuhan strain E protein comprises three domains: a short hydrophilic N-terminal domain (E-NTD, residues 1–7), a hydrophobic transmembrane domain (TMD, residues 8–38), and a long hydrophilic carboxyl terminus (C-terminal domain, E-CTD, residues 39–75) [9]. The amino acid sequence between the two viruses differs by only three substitutions (T55S, V56F, E69R) and one deletion (G70del) on the E-CTD. The sequences of the TMD of SARS-CoV and SARS-CoV-2 are identical and highly hydrophobic because of the abundance of valine and leucine residues. The total net charge of the protein is zero [10,11].

The transmembrane M protein, which comprises 222 amino acids, is the most abundant protein in the SARS-CoV-2 envelope and shares 90% homology with the SARS-CoV membrane protein [12]. M plays a central role in viral morphogenesis and interacts with other structural proteins, directing their localization in the endoplasmic reticulum and Golgi [13,14]. More specifically, the membrane protein drives nucleocapsid incorporation and, together with E, regulates intracellular trafficking of the spike protein to allow virion assembly. M protein also inhibits interferon (IFN-I) signalling by interacting with MDA5, TRAF3, and IKK_ε_, and via degradation of TBK1. It inhibits STAT1 phosphorylation, impairs MAVS activation, and interferes with nuclear translocation of IRF3 [2]. The M protein consists of three structural domains: an N-terminal domain with three transmembrane helices (TMH1, TMH2, and TMH3, residues 19–105), a juxta membrane hinge region (residues 106–116), and an inward-facing C-terminal β-sheet sandwich domain (M-CTD, residues 117–201). In addition, extravirion residues in the N-terminal domain (M-NTD) residues 1–8, and intravirion residues 207–222 are disordered [13]. The M protein is structurally similar to ORF3a, but unlike the latter, it has a different function from the ion channel [15].

The N protein of SARS-CoV-2 encapsulates viral RNA into long-stranded ribonucleocapsid complexes, and it is essential for viral assembly through the interaction with M protein [16,17]. By sequestering G3BP1, N impairs stress granule formation, reduces RIG-I activation, and compromises MAVS and IRF3 activation, in addition, it counteracts nuclear translocation of STAT1 and STAT2 [2].

The N protein comprises 419 amino acids, and its structure, like that of other coronaviruses, can be divided into intrinsically disordered regions (IDRs) and conserved structural regions [16,18]. The IDRs are divided into three different modules: the N-arm (residues 1–43), a central linker region (LKR; residues 175–254), which is a phosphorylation site that is rich in Ser/Arg, and a C-tail (residues 365–419). The conserved structural regions include an N-terminal domain (N-NTD, residues 44–174) connected by the LKR to a C-terminal domain (N-CTD, residues 255–364). These two regions are flanked by the N-arm and the C-tail [16]. The N protein has been inferred to form dimers and higher-order oligomers even without RNA [19].

While mutations in the S protein are known to influence SARS-CoV-2 evolution and fitness, mutations in non-spike proteins may also play a pivotal role, either individually or through their interactions, potentially conferring advantages to the virus [20]. To assess the impact of genomic changes, it is crucial to also consider mutations in other structural proteins, including the E, M, and N proteins.

In this study, a mutational analysis of these three structural proteins of SARS-CoV-2 in isolates from routine genomic surveillance samples collected in Verona, Italy, between February 2022 and March 2024 was performed. Although our dataset represents a subset of the Italian sequencing efforts, the results from AOUI Hospital of Verona provide added value by offering a continuous, hospital-based view of viral evolution between February 2022 and March 2024. This setting allows us to observe regional mutational dynamics during the transition between each variant, complementing national and global datasets. The trends observed in Verona are consistent with broader regional and national patterns (as observed using CoV-Spectrum tool), supporting the representativeness of our cohort while also highlighting local fluctuations that may be masked in aggregated large-scale analyses. Although several studies have catalogued SARS-CoV-2 mutations on a global scale, detailed, long-term analyses of structural protein mutations within clearly defined regional populations are still lacking. Large international datasets often lack continuous sampling from the same clinical setting, making it difficult to capture short-term shifts and co-emergence patterns. By integrating a hospital-based dataset with tightly controlled temporal resolution, this study addresses this gap and allows the identification of mutational trends that might otherwise remain undetected. Additionally, we correlate published findings with the temporal patterns identified in our dataset. This analysis focuses on the structural M, N, and E viral proteins, investigating the prevalence and potential impact of their mutations over time in Verona and evaluating how local temporal trends align with global mutational dynamics. This study aims to enhance understanding of the role of structural protein mutations in SARS-CoV-2 evolution, proposing a trend-based classification of these substitutions and providing insights into their possible epidemiological and functional implications.

## 2. Materials and Methods

### 2.1. Sample Collection, RNA Extraction, and Quantification

Nasopharyngeal swab samples (Copan, Brescia, Italy) were collected from healthcare workers and patients of AOUI Hospital of Verona, Italy, as part of SARS-CoV-2 sequencing surveillance as previously described [21]. RNA extraction was performed on the Nimbus semi-automated instrument (Seegene, Seoul, Republic of Korea) following the manufacturer’s protocol. Quantitative RT-PCR was performed on the Bio-Rad CFX 96 System (Hercules, CA, USA), using the Allplex SARS-CoV-2 Assay (Seegene, Seoul, Republic of Korea) following the manufacturer’s protocol. Only samples with a cycle threshold (Ct) below 32 were included in the study. The selection of samples for sequencing followed quality-based criteria, as Ct values are known to correlate with viral load and sequencing performance. On this basis, we analyzed a representative subset of SARS-CoV-2–positive samples from the AOUI Hospital of Verona, ensuring reliable genomic data generation. The sample distribution for each month is reported in Supplementary Table S1.

### 2.2. Next-Generation Sequencing (NGS)

Library preparation was carried out with COVIDSeq Assay (Illumina, San Diego, CA, USA) following the manufacturer’s instructions. Briefly, the extracted viral RNA was first used as input for reverse transcription to generate cDNA. The resulting cDNA underwent multiplex PCR using the ARTIC v4 and v4.1 primer pools. After amplification, the individual amplicons were combined and subjected to enzymatic clean-up to remove residual primers and reaction components. The purified amplicon pool was then processed through a series of enzymatic steps that enabled end-repair and the incorporation of Illumina sequencing adapters together with dual indexes specific for each sample, resulting in libraries ready for sequencing. These libraries were then normalized and pooled according to the assay workflow before undergoing standard quality assessment recommended by the manufacturer. Samples were sequenced with the MiSeq instrument (Illumina, San Diego, CA, USA) in paired-end mode (2 × 150 bp) with V3 chemistry.

### 2.3. Bioinformatic Analysis

The sequence analysis used a custom pipeline using SAMtools v1.21 [22] and Minimap2 v2.28 [23] on Linux bash. The SARS-CoV-2 reference genome (NC_045512.2) was used for sequence-based analysis. As the protocol was amplicon-based, mutations at primer binding sites may introduce primer bias, leading to uneven amplification and potentially resulting in variable coverage or amplicon dropouts. To address this issue, strict parameters were employed during the assembly stage: minimum depth 50, minimum mapping quality 30, and maximum call fraction 0.9. Below these constraints, no base was called. Mutations and lineages were identified through Pangolin COVID-19 Lineage Assigner [24] and the Nextclade tool from Nextstrain [25]. Further control of the sample reads distribution was performed manually using the Integrative Genomics Viewer (IGV) v2.17.3 tool [26] and FastQC v0.12.0 [27]. The obtained sequences were submitted to the national Italian COVID-19 Genomic (I-Co-Gen) platform, where the SARS-CoV-2 RECoVERY software v4.0, developed by the Istituto Superiore di Sanità (ISS), automatically performed an additional data quality control. Finally, all SARS-CoV-2 genome sequences were submitted to GISAID (Global Initiative on Sharing Avian Influenza Data, https://gisaid.org/ (accessed on 25 September 2025) [28] in the context of the Italian national surveillance project. Mutational analysis was curated manually based on literature reviews. Statistical analysis was conducted with Python v3.8.10 pandas module and visualized with Seaborn v.0.13.2 [29] and Matplotlib 3.10.6 [30]. All 3D structures were visualized with ChimeraX [31] entry 5X29 from Protein Data Bank for E protein (with template number 5x29.1.A), 8CYK for M protein, and the model obtained from Viro3D for N protein (GenBank accession number QHD43423.2).

## 3. Results and Discussion

A total of 1333 SARS-CoV-2 sequences from samples collected at the AOUI Hospital of Verona from February 2022 to March 2024 were analyzed. Only mutations with a frequency exceeding 1% of the total were included in the analysis. This threshold was chosen arbitrarily, but in line with recommendations for genomic surveillance of SARS-CoV-2. A relative proportion of around 1% is suggested as a reasonable lower bound for reliably detecting and monitoring circulating variants in a surveillance context [32]. However, this choice may have excluded rare variants present below 1%. Only 5 mutations (E:V52I, M:T30A, N:S33A, N:T334I, and N:T379I) fall within 0.5–1% prevalence range, representing 2.8% of the total mutations identified in this study.

Although these variants may be of biological interest, they were considered below our threshold for robust statistical and epidemiological relevance. The mutations were classified based on their trend during the investigated period as fixed, emerging, and transient.

**Figure 1:**
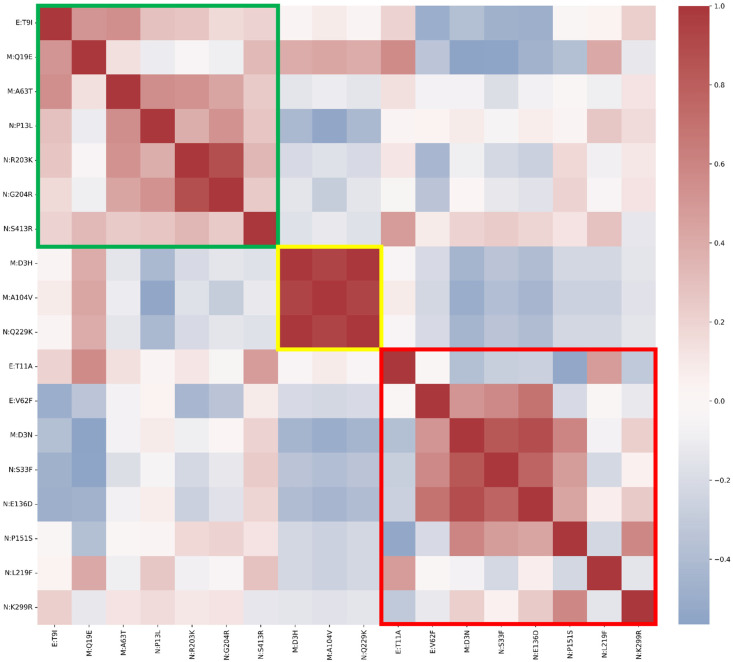
Spearman’s correlation heatmap matrix for all E, M, and N substitutions, grouped according to the classification proposed in this study. The coloured boxes highlight the specific correlations between substitutions belonging to the same group: green, fixed; yellow, emerging; and red, transient mutations.

Fixed mutations were defined as substitutions that were consistently detected throughout the study period without significant fluctuations in frequency, i.e., with a normalized monthly prevalence ≥ 80% across the entire observation window, allowing for no more than two consecutive months below this threshold. Seven mutations met these criteria and were classified as fixed, reflecting their stable acquisition within the viral population. Three mutations were classified as emerging, defined as substitutions that appeared later and persisted until the end of the investigated period (i.e., substitutions with a relative monthly prevalence of 0% for at least the first month, followed by a sustained increase that eventually exceeds 90%). We defined a mutation as transient when it appeared and subsequently disappeared during the study period, characterized by a single distinct prevalence peak (specifically, when its relative monthly prevalence exceeded 1% for at least two consecutive months and then fell below 1% for more than two consecutive months). Eight mutations were classified in this category. The correlations between substitutions of each group are shown with a Spearman correlation heatmap in Figure 1.

**Figure 2:**
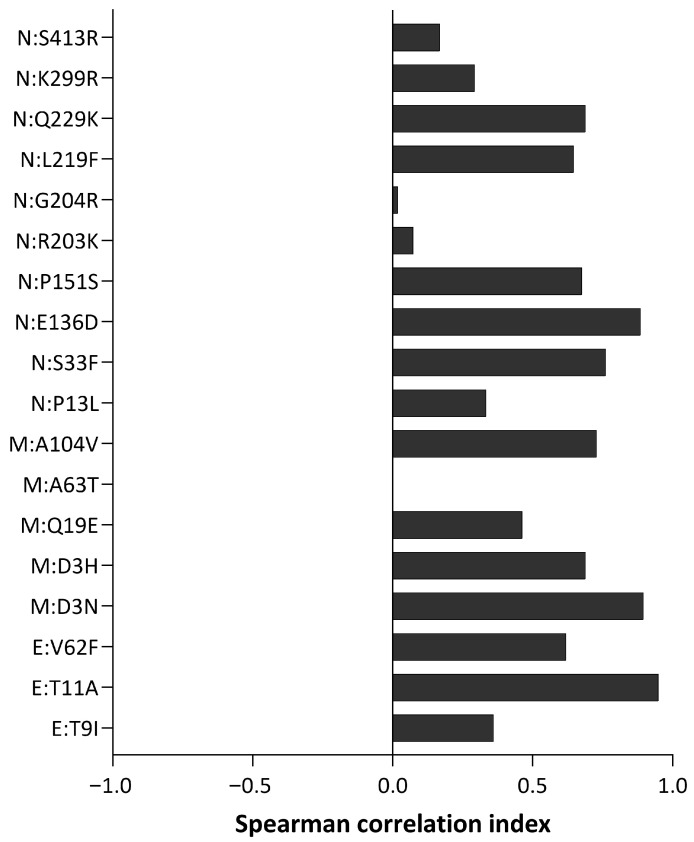
Spearman’s correlation index of the global trend compared to that observed in this study for each mutation. All mutations exhibited positive correlation indices, suggesting that the substitution trends observed in this study are consistent with global patterns.

Mutational trends were manually verified with CoV-Spectrum [33]. Spearman’s correlation analyses were conducted to compare the global trend of each mutation with that observed in this study. The results are shown in Figure 2. The emerging mutations M:D3N, M:A104V, and N:Q229K displayed a strong correlation, with a concomitant rise in prevalence in November 2023. However, experimental proteomic studies are needed to validate their interactions and assess their functional impact, beyond these in silico predictions.

Spearman’s correlation analysis between the mutations identified in this study and S substitutions previously reported [6] highlighted a strong temporal correlation for several of the emerging mutations. The group of correlated substitutions is shown in Supplementary Figure S1. Notably, the structural mutations in this study exhibited high correlation coefficients (>0.8) with 17 spike mutations (R21T, S50L, V127F, L216F, H245N, A246D, V445H, N450D, L452W, L455S, N481K, E484K, E554K, A570V, P621S, P681R, and P1143L), indicating that these variants tended to emerge concurrently. However, the functional interactions among these mutations and their combined effects on viral phenotype remain to be determined.

All substitutions show a positive correlation index. Three mutations, M:A63T, N:R203K and N:G204R, had values below 0.1 and were further investigated. These mutations were classified as fixed, and their low correlation seems to be caused by artefacts, as variability in the local trends is minimal.

### 3.1. Envelope Mutations

**Figure 3:**
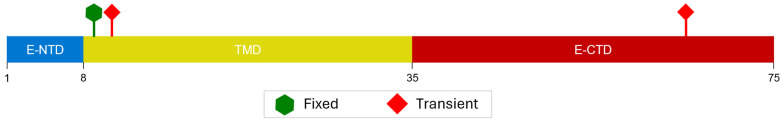
Mutation distribution in the E protein. Each mutation is coloured differently according to the following classification: green, fixed; red, transient. E-NTD: E protein N-terminal domain; TMD: hydrophobic transmembrane domain; E-CTD: E protein C-terminal domain.

**Figure 4:**
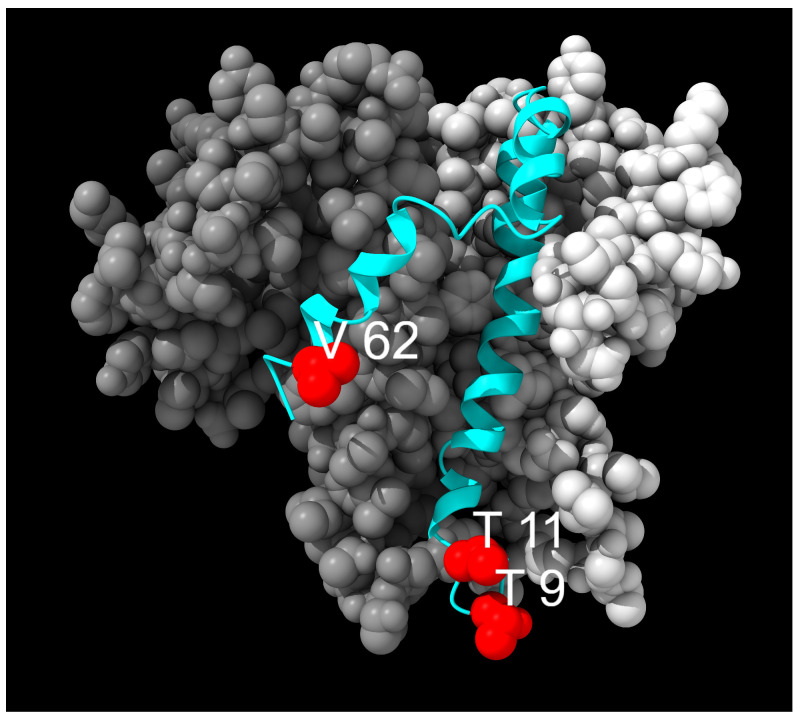
Distribution of mutation sites on the E protein organized in a homopentamer (5X26). Mutated residues are highlighted in red. One chain is displayed as a cyan ribbon, and the other chains are displayed using grey space-filling atoms.

Sixteen mutations were identified in the E protein, most of which occurred at frequencies below 1%. However, three mutations were observed at higher occurrences: T9I (95.8% of total prevalence), which was classified as fixed; T11A (33.46%), and V62F (1.58%), which were classified as transient. Mutations identified within the E protein are shown in Figure 3 and Figure 4.

The first and most prevalent mutation, T9I, was found in almost all sequenced genomes. This mutation is advantageous because it is associated with an increased resistance to autophagy, possibly by enhancing E localization to autophagosomes and interacting with autophagosome-associated proteins SNX12, ST12, TMEM87b, and ABCG2. T9I is predicted to have a Gibbs free energy (ΔΔG) of 0.190 kcal/mol and is reported to have a stabilizing effect on the E protein [34]. This mutation is also found in bat coronaviruses suggesting a SARS-CoV-2 evolutionary adaptation to the host [35].

T11A, a transient mutation, emerged in November 2022 and persisted for almost a year. By November 2023, its prevalence began to decline, and it disappeared completely after February 2024. Notably, T11A has been associated with reduced replication and attenuated virulence of SARS-CoV-2. It has been described as a dominant-negative mutation, since its expression leads to reduced cell death and cytokine release [36,37]. A similar phenomenon with the same outcome was observed with recurring deletions of the ORF8 gene [38,39]. Nevertheless, the T11A mutation determined a greater viral fitness until the emergence of new variants carrying the wild type. 

**Figure 5:**
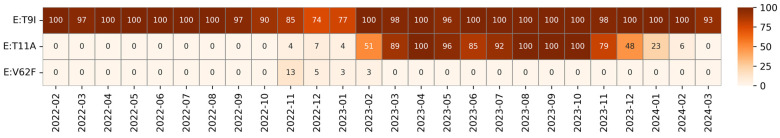
Heatmap of the relative monthly prevalence of the envelope mutations from February 2022 to March 2024. Each cell reports the percentage of sequences carrying a given mutation in a specific month, with correlations represented on a diverging colour scale as reported on the right side of the heatmap.

V62F, another transient mutation, was observed between November 2022 and February 2023, albeit at lower total prevalence compared with the other mutations. This substitution is located in the E-CTD and its impact remains to be elucidated. A single study reports that this mutation could negatively impact E protein stabilization, with a ΔΔG of 3.04 kcal/mol. This elevated ΔΔG value indicates that this substitution is highly destabilizing for the protein, suggesting that the mutation markedly disrupts its stability. This may explain both its low frequency in our dataset and its rapid disappearance after its first detection [40]. While such destabilization could affect viral fitness or pathogenicity, targeted functional assays and further structural analyses are needed to confirm this hypothesis [41]. Table 1 summarizes the mutations of the E protein, and their trends are presented in Figure 5.

### 3.2. Membrane Protein Mutations

**Figure 6:**
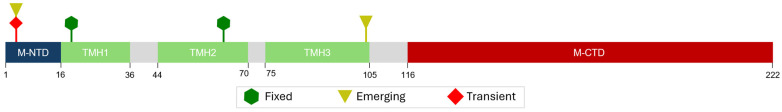
Distribution of mutation on the M protein. Each mutation is coloured differently according to the following classification: green, fixed; yellow, emerging; and red, transient. M-NTD: M protein N-terminal domain; TMH1: transmembrane helix 1; TMH2: transmembrane helix 2; TMH3: transmembrane helix 3; M-CTD: M protein C-terminal domain.

**Figure 7:**
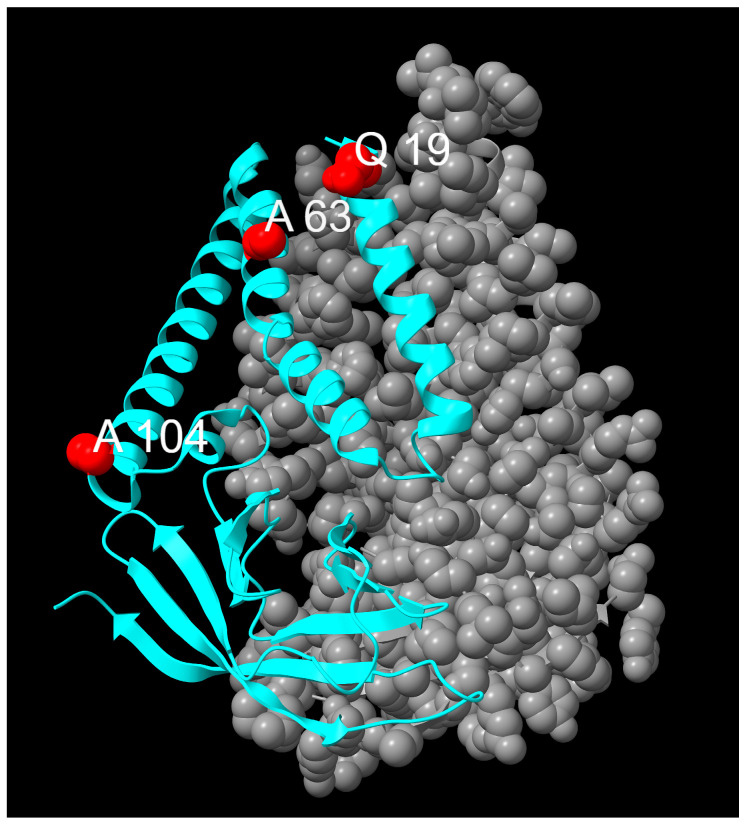
Distribution of mutation sites on the M protein (residues 17–204, 8CTK). Mutations are highlighted in red. One chain is displayed as a cyan ribbon, the other chain of the dimer is represented in grey.

Within the M gene, 33 mutations were observed, of which only five were above 1% total frequency: Q19E (76.59%) and A63T (99.77%), classified as fixed; D3H (10.5%) and A104V (11.18%), classified as emerging; and D3N (38.48%), classified as transient. Mutations detected in the M protein are displayed in Figure 6 and Figure 7.

Q19E and A63T were both found in almost all sequences. Their localization is upstream of the TMH1 of the N-terminal region and in the TMH2, respectively [8]. The Q19E mutation leads to a polarity and charge shift, replacing glutamine (polar, uncharged) with glutamic acid (polar, negatively charged) at physiological pH. To the best of our knowledge, no studies have reported to the impact of this mutation in SARS-CoV-2, even though the tryptophan residue in position 20 seems to play a pivotal role in M dimer interaction and stabilization [42]. The Q19E mutation was not initially categorized as fixed, but further analyses using less stringent parameters (minimum depth, 30) revealed the presence of this mutation at above 90% prevalence for the whole period, thus meeting the criteria for fixed mutations.

The A63T mutation also results in a change in amino acid polarity, from alanine (non-polar, hydrophobic) to threonine (polar, hydrophilic), which may influence the stabilization of the M protein dimer due to its proximity to a key residue, V66 [42].

Stability predictions for these two fixed mutations yield mixed results, as different computational tools classify them as either stabilizing or destabilizing for the M protein [43]. Nevertheless, their consistent presence in nearly all sequences in our dataset suggests that they may confer a selective advantage to the virus. However, this interpretation remains speculative, and dedicated in vitro studies are required to validate its functional impact.

From May 2022 to July 2023, the transient D3N substitution was observed, mainly in lineages BA.5.1, BQ.1.1, and BA.5.2, and their sublineages [33]. This substitution is located in the N-terminal disordered region and results in a change in charge, replacing aspartic acid (polar, negatively charged) with asparagine (polar, uncharged). Such variation could result in an N-myristoylation site at the 3–8 position, as reported by Jakhmola and colleagues [44]. N-myristoylation is a covalent, irreversible post-translational addition of a myristic acid molecule, a 14-carbon fatty acid, to the amino group of the N-terminal of a protein by the enzyme N-myristoyltransferase. N-terminal myristoylation of certain viral and host proteins plays a key role in determining their subcellular distribution and in modulating interactions with membranes and other proteins [45,46,47]. This mutation could have been favourable for BA.5.1, BA.5.2, and BQ.1.1 and their sublineages, until the emergence of new XBB variants. 

D3H and A104V, which were classified as emerging mutations, were observed in our sequences from November 2023, showing a rapid increase in frequency, even though D3H was already present in a few sequences in September 2023. As mentioned above, the substitution of the third amino acid with a charge change (from negatively to positively charged) could increase viral fitness [44,47]. It is noteworthy that a mutation at the same residue, which was previously observed and then later declined (transient mutation D3N), reappeared in November 2023 with the emergence of new, more favourable lineage descendants of BA.5.1, including JN.1 and its sublineages. This suggests the potential importance of residue D3, and further research is required.

The A104V substitution, located in the TMH3 of the N-terminal region, was also mainly found in the JN.1 and BA.2.86 lineages. This mutation involves the replacement of an alanine (hydrophobic), with a valine, which is also hydrophobic. This mutation does not alter the polarity of the residue.

**Figure 8:**
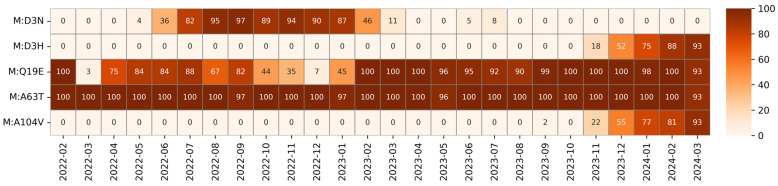
Heatmap of the relative monthly prevalence of the membrane mutations from February 2022 to March 2024. Each cell reports the percentage of sequences carrying a given mutation in a specific month, with correlations represented on a diverging colour scale as reported on the right side of the heatmap.

However, according to predictions made by SWISS-MODEL [48], both the D3H and A104V mutations and A63T show no significant modification in protein structure [42,49]. Table 2 summarizes the mutations of the membrane protein, and their trends are presented in Figure 8.

### 3.3. Nucleocapsid Mutations

**Figure 9:**
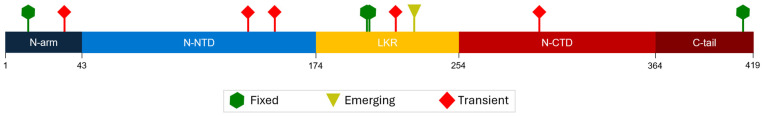
Mutation distribution on the N protein. Each mutation is coloured differently according to the following classification: green, fixed; yellow, emerging; and red, transient. N-NTD: N protein N-terminal domain; LKR: linker region; N-CTD: N protein C-terminal domain.

**Figure 10:**
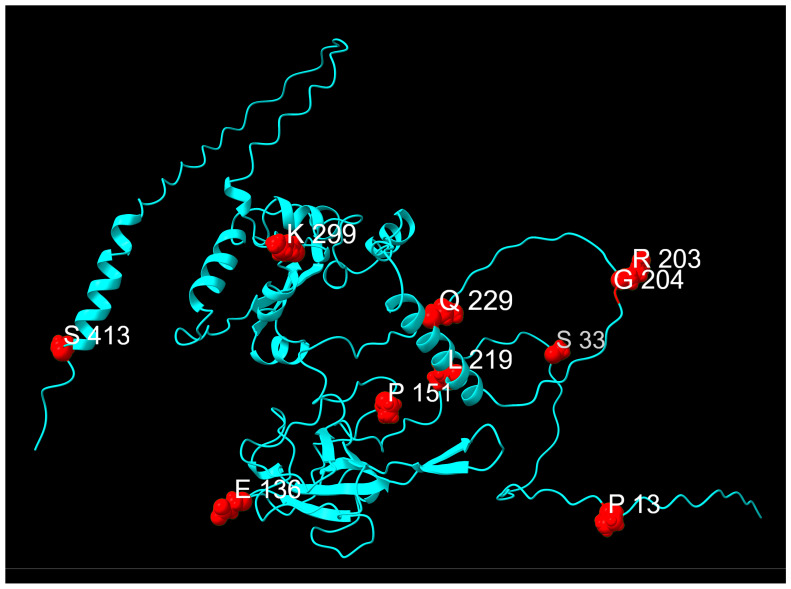
Distribution of mutation sites on the N protein. Mutation sites are highlighted in red. The protein chain is displayed as a cyan ribbon.

Of the 129 mutations detected within the N gene, ten were suitable for our analysis: P13L (99.55%), R203K (99.93%), G204R (99.85%) and S413R (99.4%), which were classified as fixed mutations; S33F (3.83%), E136D (14.83%), P151S (18.01%), L219F (1.35%), and K299R (15%) which were classified as transient mutations, and Q229K (10.58%) which was classified as an emerging mutation. Mutations identified within the N protein are shown in Figure 9 and Figure 10.

P13L is a fixed mutation that leads to structural stabilization by replacing proline (positively charged and rigid) with leucine. This substitution is associated with increased transmissibility and reduced mortality [50,51,52]. The associated mutations R203K and G204R, decrease the structural stability and flexibility of N protein, while simultaneously enhancing pathogenesis, virulence, and viral fitness, as reported both in vitro and in vivo [52,53,54,55]. The R203K mutation involves a conservative substitution between two positively charged residues (arginine and lysine), whereas G204R replaces glycine, a small residue, with arginine, a bulkier and positively charged amino acid, potentially affecting conformational dynamics. The fixed mutation S413R is located in the C-tail and appears to maintain the stability of the N protein structure without affecting its dimerization [52,56].

Several transient mutations were found during our analysis period. The S33F mutation, in the N-arm of the IDR, was found in BF.7 and BA.5.21 sublineages mainly from July 2022 to March 2023, and completely disappeared after July 2023. To the best of our knowledge, no data are available in the literature about this substitution. Nevertheless, the substitution from a polar to a hydrophobic residue (serine to phenylalanine) does not confer a selective advantage, as evidenced by its lack of persistence in subsequent viral lineages.

E136D mutation was first observed in June 2022 and decreased drastically in March 2023, disappearing a few months later with the onset of the BQ.1 lineage. This mutation on the N-NTD does not alter the polarity of the residue that remains negatively charged (glutamic acid changes to aspartic acid). No structural modifications are reported for E136D, which is supported by the unchanged performance of rapid antigen tests on variants carrying this mutation [57].

The L219F mutation was detected at low frequency in EG.1 family lineages between February and June 2023. Residues A218 and L219, located on the leucine-rich segment of LKR, stabilize the structure through persistent hydrophobic interactions with residue 215 [58]. This substitution maintains hydrophobicity without altering polarity (leucine is replaced by phenylalanine) and is unlikely to have had a significant impact on viral function, as it was subsequently lost after a few months.

Substitution P151S was found at low frequency between June and October 2022. This mutation, located in the N-NTD, has been studied in vitro and does not interfere with the domain folding; however, variants carrying this mutation may benefit from reduced detection, thereby evading the immune system. The substitution of proline with serine leads to the removal of a rigid side structure (pyrrolidine ring), which is replaced by a polar and more flexible residue that is capable of forming hydrogen bonds. This modification may alter the local conformation of the N-NTD domain and could explain to the reduced immune recognition reported by Dhamotharan et al. [59]. This substitution was found predominantly in BA.4 sublineages but was maintained in other viral variants.

The transient mutation K299R, located in the N-CTD, was found only in June and July 2022 in BA.2.3.15 lineages, and, like the previously mentioned mutation, does not change the residue polarity (lysine to arginine). No studies report the effect of mutation K299R on the N protein, but it can be considered a single point mutation gained specifically by lineage BA.2.3.15, which disappeared along with the variant, likely due to the lack of any selective advantage for the virus.

**Figure 11:**
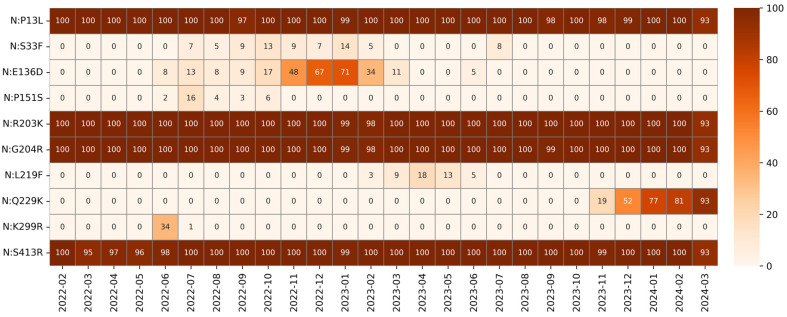
Heatmap of the relative monthly prevalence of nucleocapsid mutations from February 2022 to March 2024. Each cell reports the percentage of sequences carrying a given mutation in a specific month, with correlations represented on a diverging colour scale as reported on the right side of the heatmap.

From November 2023, with the advent of the JN.1 variant, we observed the emergence of Q229K, a mutation within the conserved leucine-rich LKR region (218–231). This region overlaps with a previously identified nuclear export signal and a peptide binder for HLA-A02:01 in regions 224–230 and 222–230, respectively [60,61]. Structure-prediction studies also confirm the presence of a helical segment in the 215–235 region which may play an important role in protein oligomerization and co-assembly with nucleic acids [58]. However, the effects of the transient substitution Q229K are unknown. This mutation was found only at low prevalence in June and July 2022, and the clinical impact is unknown. Table 3 summarizes the mutations of the nucleocapsid protein, and their trends are presented in Figure 11.

## 4. Conclusions

We performed a detailed mutational analysis of 1333 SARS-CoV-2 genomes obtained through NGS from nasopharyngeal swabs collected during epidemiological surveillance at AOUI Verona Hospital between February 2022 and March 2024. Across the structural proteins E, M, and N, a total of 178 mutations were detected, of which 18 met the inclusion threshold of <1% prevalence in our dataset. Based on their temporal patterns, substitutions were classified into fixed, emerging, and transient categories, reflecting distinct evolutionary dynamics. Functional implications were inferred primarily from published evidence and computational predictions. Additionally, several of the detected substitutions, particularly those classified as transient or of low prevalence, lack functional characterization, underscoring the need for experimental validation through both in vitro and in vivo studies.

Several limitations of this study should be acknowledged. This study is limited to a single-centre dataset, but the mutational trends observed in Verona are consistent with global evolutionary dynamics. The monocentric design may restrict the generalisability of the findings to broader or more heterogeneous populations. Monthly normalization was applied to account for variations in the sample size, but low sampling density in certain months, particularly after the termination of mandatory surveillance of healthcare personnel in March 2023, may have introduced bias and reduced statistical power. Additionally, the lack of clinical and demographic metadata further prevented analyses of potential associations between viral mutations and host-related factors such as age, vaccination status, disease severity, and comorbidities.

Although mutations in the E, M, and N genes have become established within several SARS-CoV-2 lineages, their impact is generally considered limited compared with that of spike substitutions, particularly with respect to infectivity, transmission, and immune interactions [6]. Nevertheless, minor changes affecting these structural proteins may still influence processes such as viral assembly, budding, or intracellular trafficking, and could therefore contribute to viral fitness in more subtle ways. These considerations remain speculative, and in vitro specific studies will be essential to validate their functional relevance and clarify their potential role in viral entry, replication, or spread.

Despite these constraints, the mutational trends identified in this study are broadly consistent with patterns reported in national and international genomic surveillance databases [33,62,63,64]. This highlights the importance of genomic surveillance through sequence analysis, particularly in highly exposed cohorts and at-risk populations, to capture relevant viral dynamics and emerging variants [62,63,64]. Future studies should integrate multicentre datasets, different cohorts, comprehensive metadata, and experimental assays (including structural modelling, replication studies, and antigenic characterization) to confirm phenotypic effects and optimize public health surveillance. Overall, our findings enhance the understanding of mutations in SARS-CoV-2 structural proteins beyond the spike protein and highlight the importance of sustained genomic monitoring to anticipate the emergence of novel variants with epidemiological or clinical relevance.

## Figures and Tables

**Table 1:** List and features of identified E protein mutations. The table reports the change in the polarity of the residues involved in mutations, as well as their domain location, the overall functional involvement, and the classification given in this study. E: envelope; TMD: hydrophobic transmembrane domain; E-CTD: E protein C-terminal domain.

E Mutations	Wild Type Polarity	Mutated Polarity	Domain	Involvement	Classification
T9I	Polar	Hydrophobic	TMD	Autophagy resistance [35]	Fixed
T11A	Polar	Hydrophobic	TMD	Reduced pathogenicity [36]	Transient
V62F	Hydrophobic	Hydrophobic	E-CTD	Unknown	Transient

**Table 2:** List and features of identified membrane mutations. The table reports the change in the polarity of the residues involved in mutations, as well as its domain location, the overall functional involvement and the classification given in this study. M: membrane, M-NTD: M protein N-terminal domain; TMH1: transmembrane helix 1; TMH2: transmembrane helix 2; TMH3: transmembrane helix 3.

M Mutation	Wild Type Polarity	Mutated Polarity	Domain	Involvement	Classification
D3N	Negative	Polar	M-NTD	Protein interaction modulation [44]	Transient
D3H	Negative	Positive	M-NTD	Protein interaction modulation [44]	Emerging
Q19E	Polar	Negative	TMH1	Protein stabilization [42]	Fixed
A63T	Hydrophobic	Polar	TMH2	Protein stabilization [42]	Fixed
A104V	Hydrophobic	Hydrophobic	TMH3	Unknown	Emerging

**Table 3:** List and features of identified nucleocapsid mutations. The table reports the change in the polarity of the residue involved in mutations, as well as its domain location, the overall functional involvement, and the classification given in this study. N: nucleocapsid; N-NTD: N protein N-terminal domain; LKR: linker region; N-CTD: N protein C-terminal domain.

N Mutation	Wild Type Polarity	Mutated Polarity	Domain	Involvement	Classification
P13L	Proline	Hydrophobic	N-arm	Protein stabilization [52]	Fixed
S33F	Polar	Hydrophobic	N-arm	Unknown	Transient
E136D	Negative	Negative	N-NTD	Unknown	Transient
P151S	Proline	Polar	N-NTD	Immune evasion [59]	Transient
R203K	Positive	Positive	LKR	Increased pathogenicity [53]	Fixed
G204R	Glycine	Positive	LKR	Increased pathogenicity [53]	Fixed
L219F	Hydrophobic	Hydrophobic	LKR	Enhanced protein stability [58]	Transient
Q229K	Polar	Positive	LKR	Unknown	Emerging
K299R	Positive	Positive	N-CTD	Unknown	Transient
S413R	Polar	Positive	C-tail	Protein stabilization [52]	Fixed

## Data Availability

All sequences used in this study are available in the GISAID database at https://gisaid.org/ as follows: hCoV-19/Italy/VEN-AOUIVR-*Sample_ID*_VR/*year* (accessed on 25 September 2025).

## Supplementary Materials

The following supporting information can be downloaded at: https://www.mdpi.com/article/10.3390/idr17060150/s1, Figure S1: Spearman correlation matrix between monthly prevalence values of SARS-CoV-2 M, E, and N mutations and Spike mutations; Table S1: Monthly distribution of sequences.

## Abbreviations

The following abbreviations are used in this manuscript:

AOUI	Azienda Ospedaliera Universitaria Integrata
CTD	C-terminal Domain
E	Envelope
ER	Endoplasmic Reticulum
GISAID	Global Initiative on Sharing Avian Influenza Data
IDR	Intrinsically Disordered Region
IFN	Interferon
IGV	Integrative Genomics Viewer
ISS	Istituto Superiore di Sanità
LKR	Linker Region
M	Membrane
N	Nucleocapsid
NGS	Next-Generation Sequencing
NSP	Non-structural protein
NTD	N-terminal Domain
ORF	Open Reading Frame
PDB	Protein Data Bank
S	Spike
SARS-CoV	Severe Acute Respiratory Syndrome Coronavirus
TMD	Transmembrane Domain
TMH	Transmembrane Helix
UOC	Unità Operativa Complessa
WHO	World Health Organization
