# Supplementary material for: Beyond the Spike Glycoprotein: Mutational Signatures in SARS-CoV-2 Structural Proteins
Source: Infect Dis Rep. 2025 Dec 18;17(6):150. doi: 10.3390/idr17060150 (PMC12733084; doi:10.3390/idr17060150)
Supplement: Supplementary file 1 [file idr-17-00150-s001.zip › idr-3926794-supplementary-Proofreading Done.pdf]

Year	Month	# of Sequences
2022	Feb	1
	Mar	37
	Apr	72
	May	51
	Jun	50
	Jul	76
	Aug	100
	Sep	33
	Oct	71
	Nov	102
	Dec	42
2023	Jan	78
	Feb	65
	Mar	46
	Apr	33
	May	23
	Jun	20
	Jul	12
	Aug	20
	Sep	85
	Oct	32
	Nov	103
	Dec	91
2024	Jan	60
	Feb	16
	Mar	14

Supplementary Table S1. Monthly distribution of sequences. The table reports the monthly distribution of sequences, from February 2022 to March 2023, where each entry reflects the number of samples collected in that month.

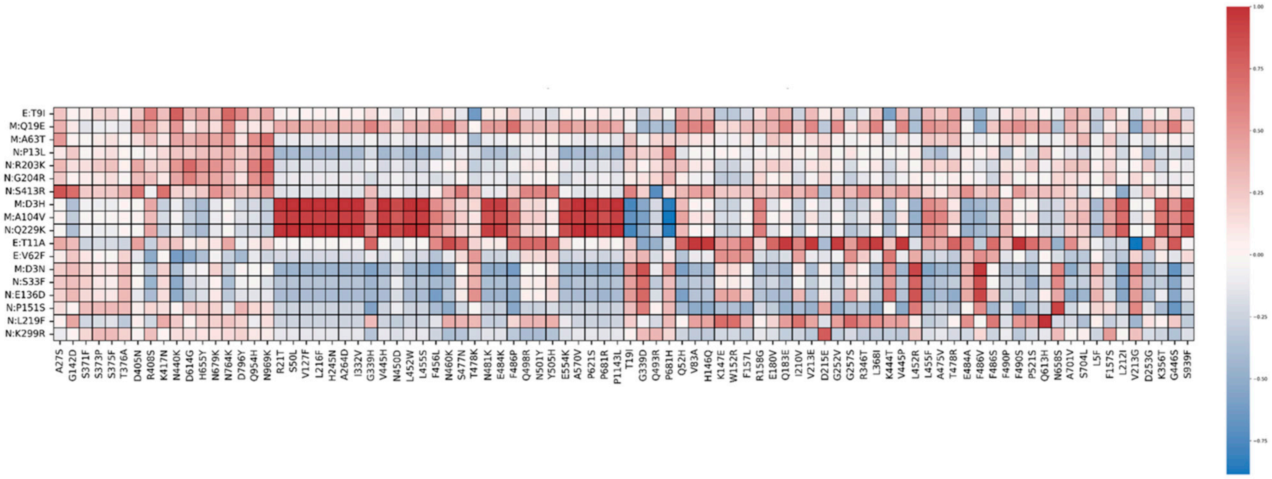

Supplementary Figure S1. Spearman correlation matrix between monthly prevalence values of SARS-CoV-2 M, E and N mutations and Spike mutations. The heatmap displays the full correlation matrix, with correlations represented on a diverging color scale as reported on the right side of the heatmap.
