# Supplementary figures and images for: Beyond the Spike Glycoprotein: Mutational Signatures in SARS-CoV-2 Structural Proteins
Source: Infect Dis Rep. 2025 Dec 18;17(6):150. doi: 10.3390/idr17060150 (PMC12733084; doi:10.3390/idr17060150)

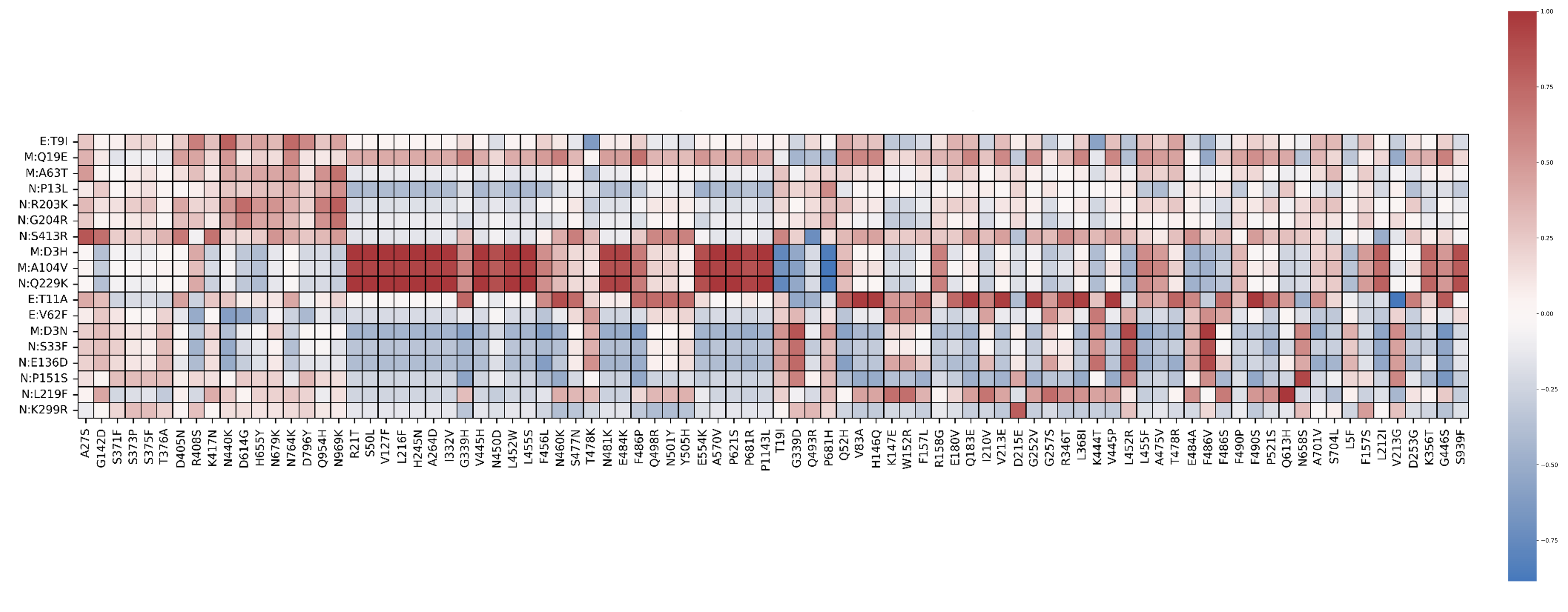

Supplement: Supplementary file 1 [file idr-17-00150-s001.zip › idr-3926794-Supplementary Figure S1.png]
